# Diabetes Remission After Bariatric Surgery: A 10-Year Follow-Up Study

**DOI:** 10.1007/s11695-024-07592-9

**Published:** 2024-12-23

**Authors:** Inês Meira, João Menino, Patrícia Ferreira, Ana Rita Leite, Juliana Gonçalves, Helena Urbano Ferreira, Sara Ribeiro, Telma Moreno, Diana Festas Silva, Jorge Pedro, Ana Varela, Selma Souto, Paula Freitas, Eduardo Lima da Costa, Joana Queirós, CRIO Group

**Affiliations:** 1 Serviço de Endocrinologia, Diabetes E Metabolismo, Centro Hospitalar Universitário de São João, Porto, Portugal; 2 Faculdade de Medicina da, Universidade Do Porto, Porto, Portugal; 3 Hospital Lusíadas, Porto, Portugal; 4 Centro de Responsabilidade Integrada de Ob, Porto, Portugal

**Keywords:** Bariatric surgery, Obesity, Type 2 Diabetes

## Abstract

**Introduction:**

Treatment of type 2 diabetes (T2DM) in patients with obesity can be challenging. Metabolic and bariatric surgery (MBS) has shown promising results in improving glycemic control and even achieving remission in T2DM patients with obesity. However, the durability of glycemic improvements in T2DM patients following MBS remains insufficiently studied.

**Aim:**

Determine the incidence of durable remission and relapse of T2DM rates 10 years after MBS, characterize the glycemic profile after surgery, and identify factors predicting persistent remission of T2DM.

**Methods:**

Retrospective observational study of T2DM patients undergoing MBS between 2010 and 2013. Clinical and analytical assessments were performed preoperatively, at 2- and at 10-years postoperatively. Paired t-tests, Wilcoxon-signed-rank and McNemar tests were used to assess the differences in the metabolic status during the follow-up. Logistic regression models were used to identify predictors of T2DM remission.

**Results:**

Ninety-five patients were included (mean age 48.8 ± 9.1 years, mean HbA1c 7.0 ± 1.5%). Ten years after surgery, the rate of complete T2DM remission was 31%, partial remission was 15%, and late recurrence after initial remission was 24%. Patients with lower HbA1c (OR = 0.50; *p* = 0.05) and taking fewer antidiabetic drugs (OR = 0.31; *p* = 0.01) preoperatively were more likely to maintain long-term remission. Ten years post-MBS, patients maintained lower fasting plasma glucose (*p* < 0.001), HbA1c (*p* < 0.001), number of antidiabetic drugs (*p* < 0.001), and insulin use (*p* < 0.001).

**Conclusion:**

MBS can induce a significant improvement and sustainable remission of T2DM. Early intervention, while patients still have a good glycemic control with a lower number of anti-diabetic drugs, is crucial to achieve long-lasting benefits and a potential "surgical cure" for T2DM.

## Introduction

Nowadays, there is consistent evidence that metabolic and bariatric surgery (MBS) is an effective strategy to improve glycemic control in diabetic patients and often leads to remission of diabetes in patients with obesity. [4–8] A systematic review concluded that type 2 diabetes was resolved in 78% and resolved or improved in 87% of patients undergoing MBS [7]. In fact, it is known that modest weight loss improves glycemia and reduces the need for glucose-lowering medications [7–9], while more substantial weight loss significantly reduces HbA1c and fasting glucose and has been shown to promote sustained diabetes remission through at least 2 years. [9, 10]

Therefore, according to the American Society for Metabolic and Bariatric Surgery and The International Federation for the Surgery of Obesity and Metabolic Disorders, MBS should be recommended for adults with T2DM and BMI ≥ 30 kg/m^2^, as a weight and glycemic management approach.[11, 12].

Several preoperative variables have been reported as predictors of T2DM remission after MBS. Younger age, a shorter duration of diabetes, and the absence of insulin therapy are associated with a higher likelihood of complete remission. Additionally, lower preoperative fasting glucose and HbA1c levels, along with higher C-peptide levels and improved pancreatic beta cell function were also described as useful predictors of T2DM remission after MBS. [1, 13–18].

The majority of the published literature supporting diabetes remission after MBS has short- and medium-term follow-up. However, the durability of glycemic improvements and the potential for long-term "cure" in T2DM patients following MBS remain insufficiently studied. Currently, few studies report long-term (≥ 5 years) diabetes remission rates, emphasizing the need for further investigation and assessment of the enduring effectiveness of these interventions. Furthermore, understanding the clinical factors that influence both the remission and the recurrence of T2DM after MBS could potentially lead to better strategies to enhance durable remission.

We hypothesize that a substantial proportion of patients undergoing MBS will sustain type 2 diabetes remission for at least 10 years and that specific preoperative factors will predict long-term remission.

The objective of this study was to determine the prevalence of durable remission and relapse of T2DM rates 10 years after MBS using standardized criteria, to characterize the glycemic profile of these patients after surgery, and to identify factors predicting persistent remission of T2DM.

## Study Design and Participants

This is a 10-year retrospective observational study conducted in a population of patients with obesity (BMI ≥ 35 kg/m^2^) followed by the Multidisciplinary Group for Surgical Obesity Management of our center.

A total of 909 patients underwent MBS between January 2010 and May 2013. Of this original cohort, we excluded those submitted to gastric band surgery (*n* = 272), those without follow-up visits available (*n* = 180) and those with missing information regarding diabetes status at baseline or 10 years after surgery (*n* = 18). Patients who had re-operative bariatric surgery (*n* = 12) for conversion of sleeve gastrectomy (SG) to Roux-en-Y gastric bypass (RYGB), conversion of SG to duodenal switch, or conversion of RYGB to distal gastric bypass with shortening of the common channel were also excluded. A total of 427 were eligible for inclusion in the current analysis.

For the RYGB procedure, at the time these surgeries were performed, the biliopancreatic limb length was 70 cm.

Participants were studied before surgery and data was collected two and ten years after surgery to evaluate changes in body weight and to assess metabolic status. Only patients with complete and documented baseline data who also had at least 10 years post-surgery follow-up, including data on body weight, fasting blood glucose (FBG) or HbA1c levels, and status of diabetes medications, were included in this study.

All outcome data included in the analysis were obtained from the electronic medical records from a clinical visit in our center or source documentation from the patient’s primary physician 2 (short-term) and 10 years (long-term) after surgery. Data on body weight, BMI, diabetes status, blood pressure, lipid panel, and diabetes medications were extracted from the preoperative period until the most recent follow-up.

All procedures performed in this study involving human participants were in accordance with the ethical standards of the institutional and/or national research committee and with the 1964 Helsinki Declaration and its later amendments or comparable ethical standards.

## Definition of Diabetes and Diabetes Remission

At baseline, participants were classified as having diabetes if they met any of the following criteria: (1) HbA1c ≥ 6.5% or, in the absence of this measurement, fasting glucose ≥ 126 mg/dL; (2) treatment with two or more anti-diabetic drugs; or (3) treatment with metformin and self-reported diagnosis of diabetes.

**Fig. 1:**
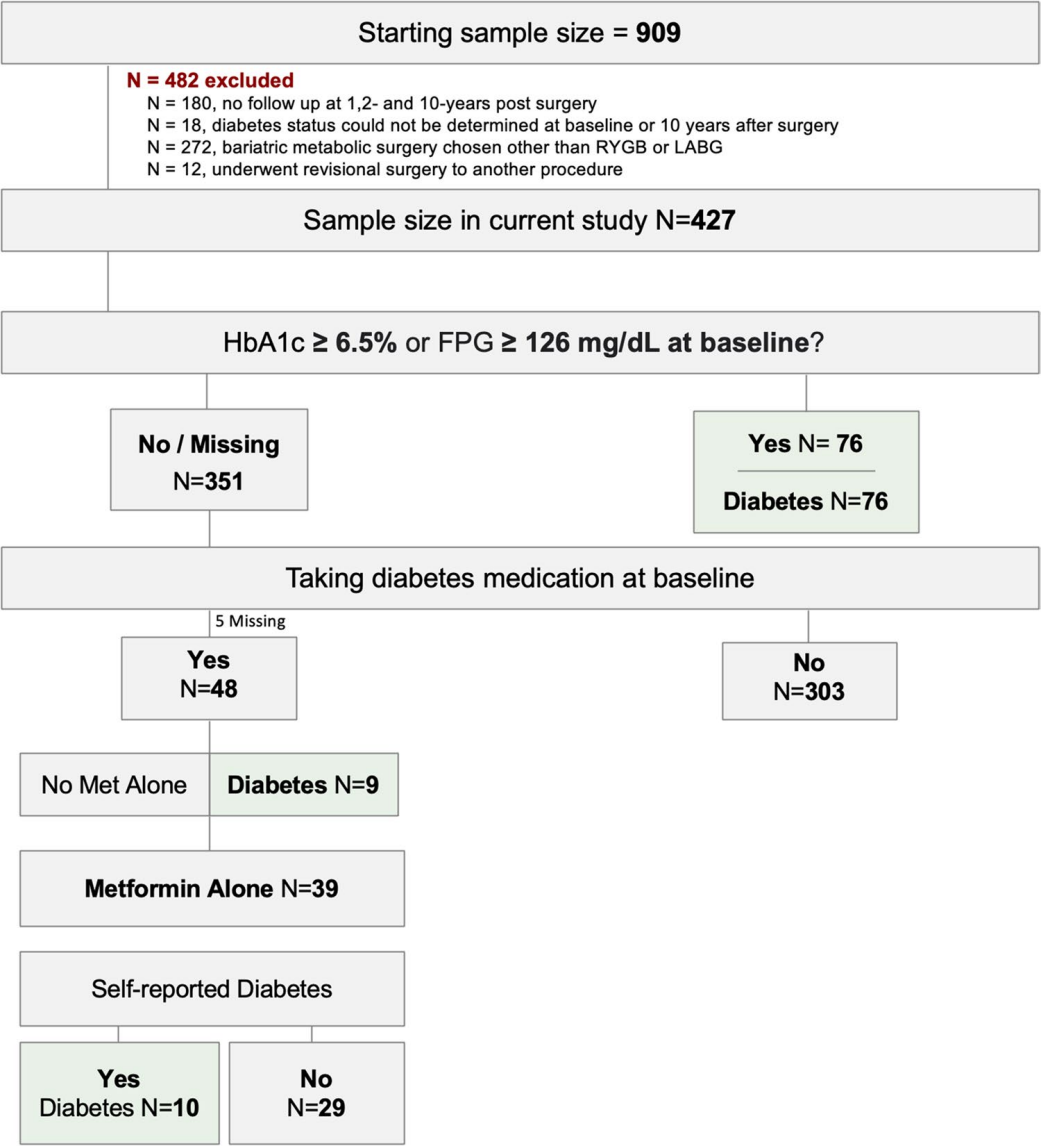
Decision tree for determining eligibility for study inclusion

Considering the widespread use of metformin for prediabetes management, weight loss, and polycystic ovary syndrome, individuals meeting all the following criteria were excluded from this analysis: patients treated exclusively with metformin (without any other anti-diabetic drug), HbA1c < 6.5%, and lacking a self-reported diagnosis of diabetes (Fig. 1).

Partial remission was defined as an HbA1c between 5.7–6.5% or, if not available, a fasting glucose 100–125 mg/dL in the absence of diabetes pharmacologic therapy. Complete remission was defined as an HbA1c < 5.7% or, if not available, a fasting glucose < 100 mg/dL in the absence of diabetes pharmacologic therapy. Diabetes recurrence was defined as an HbA1c ≥ 6.5% or need for anti-diabetic medication reintroduction after a remission period. [19]

## Statistical Analyses

Continuous variables are described as mean ± standard deviation or median (25th-75th percentiles), as appropriate, and categorical variables as proportions (percentages). Normal distribution was evaluated using Shapiro–Wilk test or skewness and kurtosis.

Paired t-tests, Wilcoxon signed rank and McNemar tests were used to assess the differences in the metabolic status during the follow-up, as appropriate.

Logistic regression analysis was performed considering diabetes remission as the primary outcome. Each predictor was initially entered in a univariate logistic regression to study its ability to independently predict diabetes remission. Only predictors that were significantly associated with remission in univariate analysis were then included in a binary logistic regression model as potential predictors of diabetes remission. We investigated possible predictors of diabetes remission with t-tests, Mann–Whitney U tests, and chi-square tests when appropriate to check for possible differences between remission and non-remission groups.

A sub-analysis among patients who did not achieve T2DM remission was conducted, to evaluate the evolution in the metabolic status during the follow-up period.

A variance inflation factor (VIF) above 5 was used to assume collinearity and to exclude variables from the regression model. Reported p values are two-tailed and p < 0.05 was considered significant. Analyses were performed with the use of SPSS Statistics 29®.

## Results

### Baseline Characteristics

We have identified 95 patients with T2DM that were enrolled in this study —81 (85.3%) submitted to RYGB, and 14 (14.7%) to SG. The mean age of the enrolled population was 48.8 ± 9.08 years old. Average weight and BMI were 114 ± 16.1 kg and 44.9 ± 6.39 kg/m^2^, respectively. The included patients had a mean pre-operative HbA1c of 7.0 ± 1.48%. At the time of the surgery, 81 (94.9%) patients were on anti-diabetic medication. Among them, 13 patients (13.7%) were on insulin therapy. Baseline characteristics of the study sample are summarized in Table 1.

**Table 1:** Clinical and biochemical characteristics of the enrolled patients before surgery (n = 95)

**Baseline features**	
Age, years	48.79 ± 9.08
Feminine sex, n (%)	80 (84%)
Weight, kg	114.8 ± 16.09
Body mass index, kg/m^2^	44.9 ± 6.39
Waist circumference, cm	124.9 ± 13.29
Hip circumference, cm	130.6 ± 15.27
Systolic blood pressure, mmHg	136.9 ± 16.37
Diastolic blood pressure, mmHg	82.8 ± 10.53
Total cholesterol, mg/dL	213.1 ± 42.39
LDL cholesterol, mg/dL	134.23 ± 37.02
Triglycerides, mg/dL	157.0 (148.96; 199.91)
Fasting plasma glucose, mg/dL	134.8 ± 49.88
HbA1C, %	7.0 ± 1.48
Fasting plasma C-peptide, ng/mL	4.4 ± 1.22
HOMA-IR	5.8 ± 2.92
Number of anti-diabetic drugs	1.37 ± 0.91
Number of patients on insulin therapy, n (%)	13 (13.7%)

Data are given as mean ± SD or median (P25; P75) for continuous variables or n (%) for categorical variables. HbA1: glycated hemoglobin; LDL: low-density lipoprotein; HOMA-IR: Homeostasis model assessment of insulin resistance

### T2DM Prevalence and Remission After Bariatric Surgery

**Fig. 2:**
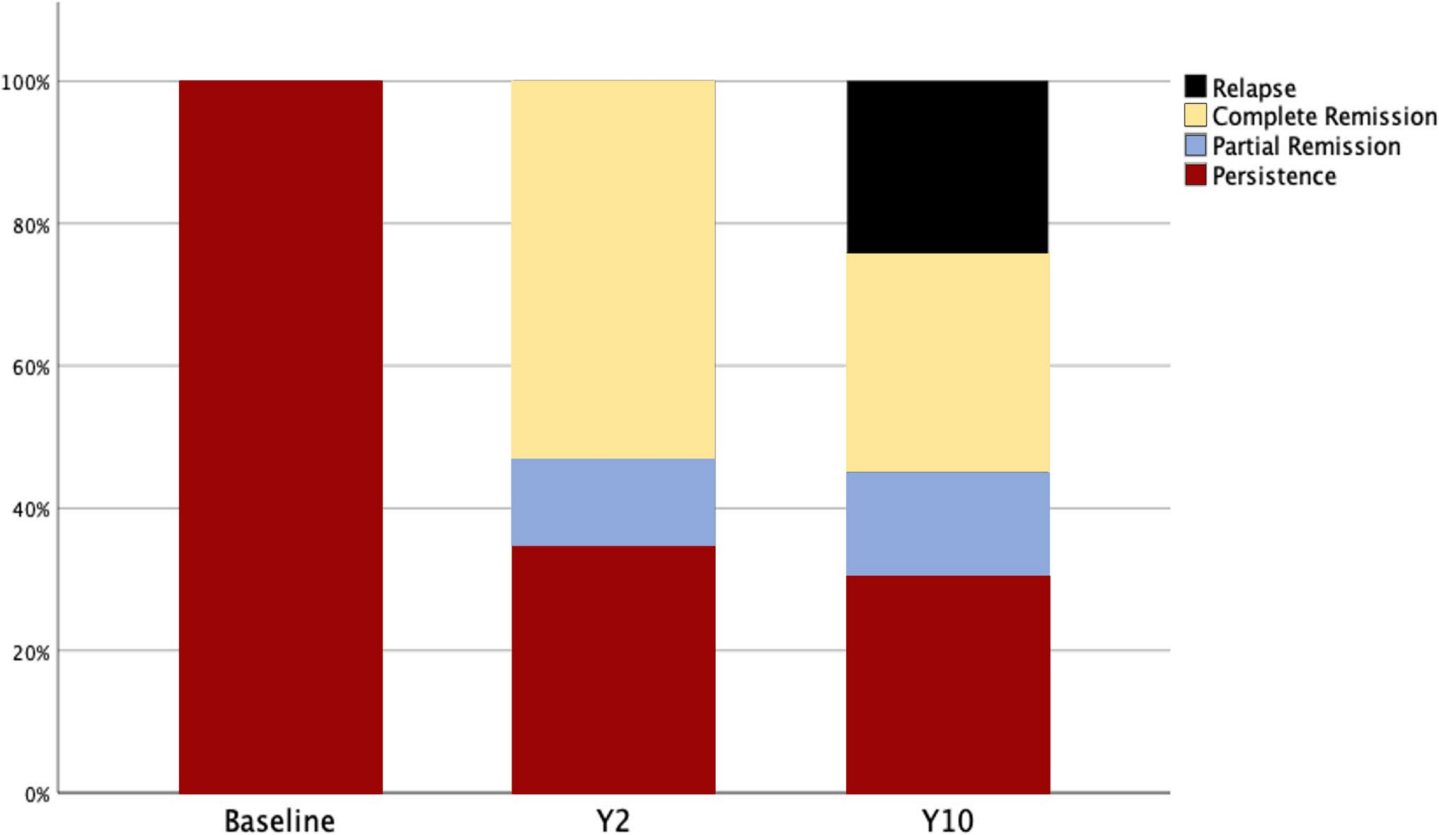
Note: This data is madatory

Figure 2 depicts the evolution of patients with diabetes throughout the follow-up. Thirty percent of the cohort achieved long-term complete remission of T2DM, and another 15% achieved partial remission. Of those patients who achieved remission at the short-term (n = 62), 23 (24%) had a recurrence of their diabetes at long-term follow-up (Table 2). The mean HbA1C decreased from 7.0% ± 1.5% to 6.1% ± 0.7% (*P* < 0.001) and the mean FBG decreased from 133.1 ± 47.2 mg/dL to 100.6 ± 21.6 mg/dL (*p* < 0.001). Overall, 10 years after MBS, patients were taking fewer numbers of anti-diabetic drugs (1.37 ± 0.09 to 0.86 ± 0.67, *p* < 0.001) and fewer patients were requiring insulin therapy (14% to 2%, *p* < 0.001)—Table 3. Ten years after MBS, 12 patients (13%) were receiving GLP-1 receptor agonist therapy.

**Table 2:** Type 2 diabetes mellitus remission and relapse rates throughout the 10 years of follow-up

	**Baseline**	**Year 2**	**Year 10**
Complete remission, n (%)	NA	50 (53%)	29 (30%)
Partial remission, n (%)	NA	12 (13%)	14 (15%)
Persistence, n (%)	NA	33 (35%)	29 (31%)
Relapse, n (%)	NA	NA	23 (24%)

NA: not applicable

**Table 3:** Long term and short-term Metabolic Profile of Diabetic Patients after Bariatric Surgery

				**p value**	
**Metabolic Parameter**	**Baseline**	**Short-term (Year 2)**	**Long-term** **(Year 10)**	Short-term vs Baseline	Long-term vs Baseline
\Fasting plasma glucose, mg/dL	133,05 ± 47.18	94.02 ± 17.03	100.59 ± 21.57	** < 0.001**	** < 0.001**
HbA1C, %	7.01 ± 1.49	5.69 ± 0.70	6.09 ± 0.69	** < 0.001**	** < 0.001**
HbA1C < 7%, n (%)	33 (35%)	7 (7%)	14 (15%)	** < 0.001**	**0.001**
Number of anti-diabetic drugs	1.37 ± 0.09	0.43 ± 0.29	0.86 ± 0.67	** < 0.001**	** < 0.001**
Insulin use, n (%)	13 (14%)	4 (4%)	2 (2%)	**0.004**	** < 0.001**

Data are given as mean ± SD for continuous variables or n (%) for categorical variables. HbA1c: glycated hemoglobin; Statistically significant p values are presented in bold

It was also conducted a sub-analysis among patients who did not achieve T2DM remission – as detailed in Table 4. Ten years after MBS, this sub-sample maintained lower HbA1c (7.55 ± 1.68 vs 6.41 ± 0.72, *p* < 0.001) and FBG (148.75 ± 57.49 vs 111.28 ± 25.35, *p* < 0.001), with a fewer proportion with need for insulin therapy (23.1% vs 3.8%, *p* < 0.001). There were no significant differences observed in the number of anti-diabetic drugs required 10 years after surgery.

**Table 4:** Long term and short-term Metabolic Profile of Diabetic Patients who did not achieved remission after Bariatric Surgery

				**p valuea**	
**Metabolic Parameter**	**Baseline**	**Short-term** **(Year 2)**	**Long-term** **(Year 10)**	Short-term **vs** Baseline	Long-term **vs** Baseline
Fasting plasma glucose, mg/dL	148.75 ± 57.49	98.56 ± 19.31	111.28 ± 25.35	** < 0.001**	** < 0.001**
HbA1C, %	7.55 ± 1.68	5.92 ± 0.80	6.41 ± 0.72	** < 0.001**	** < 0.001**
Number of anti-diabetic drugs	2 (1–2)	1 (0–1)	2 (1–2)	** < 0.001**	0.326
Insulin use, n (%)	12 (23.1%)	4 (7.7%)	2 (3.8%)	**0.008**	**0.002**

Data are given as mean ± SD for continuous variables or n (%) for categorical variables. HbA1c: glycated hemoglobin; Statistically significant p values are presented in bold

### Predictors of T2DM Remission

The comparison of preoperative features between patients that achieved remission and those who did not is shown in Table 5. Postoperative long-term diabetes remission was attained in 43 (45%) patients. These patients had lower preoperative HbA1C (mean difference of 1.25 ± 0.28%; *p* < 0.001), and lower fasting plasma glucose (mean difference of 33.2 ± 9.6 mg/dL; *p* < 0.001). Furthermore, patients on remission took fewer oral antidiabetics drugs (2 (1.0; 2.0) vs 1 (0; 1.0), *p* < 0.001) preoperatively and had shorter duration of T2DM (4 (1.0; 6.5 years vs 1 (0; 3.0) years, *p* = 0.001). Age, weight, baseline BMI, and waist circumference did not differ between both groups (*p* = 0.990, *p* = 0.550, *p* = 0.430, respectively). The calculated index of insulin resistance (HOMA-IR) and preoperative fasting plasma C-peptide levels were not statistically different (*p* = 0.46, *p* = 0.820, respectively). Most patients in this cohort were submitted to RYGB surgery, but there were more patients submitted to SG in the remission group (23.3% vs 7.7%, *p* = 0.031).

**Table 5:** Comparison of preoperative characteristics in patients with and without diabetes remission after bariatric surgery

Baseline features	**No Remission** (n = 52)	**Long-term Remission** (n = 43)	p value
Age, years	49.4 ± 7.05	48.1 ± 11.10	0.490
Feminine sex, n (%)	44 (84.6%)	36 (83.7%)	0.900
Type of surgery (RYGB vs SG)	48 (92.3%)	33 (76.7%)	**0.031**
Weight, kg	114.8 ± 15.98	114.8 ± 16.39	0.990
Body mass index, kg/m^2^	44.6 ± 6.24	45.3 ± 6.62	0.550
Waist circumference, cm	126.0 ± 13.25	123.6 ± 13.43	0.430
Fasting plasma glucose, mg/dL	150.1 ± 41.18	116.9 ± 27.58	** < 0.001**
HbA1C, %	7.6 ± 1.67	6.3 ± 0.79	** < 0.001**
Fasting plasma C-peptide, ng/mL	4.15 (3.44; 5.62)	4.47 (3.59; 5.38)	0.820
HOMA-IR	5.34 (3.94; 8.43)	5.14 (3.06; 8.02)	0.460
Diabetes duration, years	4 (1.0; 6.5)	1 (0; 3.0)	**0.001**
Number of anti-diabetic drugs	2 (1.0; 2.0)	1 (0; 1.0)	** < 0.001**
Total weight loss, %	25.1 (22.2; 23.0)	28.8 (18.2;36.5)	0.410

Data are given as mean ± SD or median [P25; P75] for continuous variables or n (%) for categorical variables. HbA1c: glycated hemoglobin; HOMA-IR: Homeostasis model assessment of insulin resistance; RYGB: Roux-en-Y Gastric Bypass; SG: sleeve gastrectomy. Statistically significant p values are presented in bold

Baseline features that achieved statistical significance between remission and non-remission groups were included in a binary logistic regression model as potential candidates to remission predictors. Fasting plasma glucose was excluded due to its collinearity with HbA1C. Data is displayed in Table 6. Lower HbA1C (OR = 0.50, 95% CI 0.24–0.98) and the number of anti-diabetic drugs (OR = 0.31, 95% CI 0.13–0.76) at baseline were significant predictors, while diabetes duration, and the type of surgery did not reach statistical significance when they were adjusted for the other included variables.

**Table 6:** Binary logistic regression analysis for prediction of remission of diabetes 10 years after bariatric surgery

	**Multivariate analysis**		
Predictors	**OR**	95% CI	p value
Preoperative HbA1c	0.50	0.24–0.98	**0.05**
Preoperative number of anti-diabetic drugs	0.31	0.13–0.76	**0.01**
Diabetes duration	0.97	0.82–1.14	0.68
Type of surgery (RYGB vs SG)	0.22	0.03–1.58	0.13

CI, confidence interval; HbA1c: glycated hemoglobin; OR: odds ratio; RYGB: Roux-en-Y Gastric Bypass; SG: sleeve gastrectomy

## Discussion

Nowadays, obesity is considered an etiologic factor of T2DM. Despite aggressive medical treatment, the natural course of this chronic disease is characterized by progression to microvascular complications such as neuropathy, chronic kidney disease, retinopathy, and cardiovascular disease. However, it is now well established that MBS strongly improves glycemic control and often leads to remission of diabetes. [1–3, 6–8, 11, 19]. In fact, MBS has been increasingly seen as a metabolic surgery and not only as a mean to reach weight reduction.

The need for long-term follow-up is one of the limiting factors in most clinical observational studies because a significant number of patients who undergo MBS are lost to follow-up 3–5 years after the surgery.

In our study, we observed a 2-year diabetes remission rate of approximately 66%, highlighting the effectiveness of MBS in short-term glucose metabolism control for patients with obesity. However, among the 62 patients with T2DM remission 2 years after surgery, 23 experienced a relapse of T2DM, aligning with other studies reporting a relapse rate between 25–35% [15, 18].

It is also crucial to identify preoperative factors that affect the durability of T2DM remission to select the most suitable patients to surgery. The most frequently identified predictors of T2DM relapse in the scientific literature were signs of diabetes severity and/or decreased pancreatic reserve, related to more advanced stages of T2DM at the time of surgery [17, 20].

Accordingly, we found that better preoperative glycemic control was associated with higher long-term remission rates. Similarly, patients treated with a lower number of anti-diabetic drugs were more likely to maintain remission of T2DM 10 years after surgery.

Although not reaching statistical significance, patients who maintained long-term T2DM remission appeared to have higher fasting plasma C-peptide levels, aligning with other studies that demonstrated preoperative beta cell function as an independent predictor of diabetes remission after MBS. Given that progressive pancreatic beta cell failure is a natural part of the course of diabetes, it can be expected that patients with less preoperative dysfunction of the beta cell have higher odds of diabetes remission after surgery. [15]

Overall, and in agreement with previous studies, these data suggest that when the main goal of surgical treatment is durable diabetes remission, a surgical intervention should be considered while patients present good glycemic control with a low number of anti-diabetic drugs, since it is likely to be more effective*.* This does not mean that MBS is not beneficial for patients with more advanced T2DM. In our study, patients who did not achieve remission maintained better glycemic control compared to the baseline. Furthermore, surgery can bring about essential enhancements in quality of life, improvement of other obesity-associated diseases (such as hypertension, hyperlipidemia, and obstructive sleep apnea), and a global reduction in cardiovascular risk, despite having a T2DM relapse. [21–23]

We showed that the likelihood of achieving diabetes remission is weight-loss independent. While certain studies have highlighted the impact of weight loss on T2DM remission after metabolic surgery [4, 15, 22], factors like diabetes duration, glycemic control, or pre-surgery insulin therapy may exert a more significant influence, potentially diluting the effect of weight loss.

In the univariate analysis, it appeared that patients who maintained long-term T2DM remission had a higher frequency of undergoing SG compared to patients who did not achieve remission 10 years after surgery. This discrepancy may be attributed to the smaller number of patients undergoing SG in our cohort, as well as the exclusion of individuals who underwent revision surgery (predominantly those who had previously undergone SG but experienced significant weight regain). Consequently, we ended up excluding cases where SG demonstrated reduced efficacy. Nevertheless, with the inclusion of other variables as independent predictive variables in the multivariate analysis, the type of surgical procedures (RYGB and SG) was no longer a significant predictor of T2DM long-term remission.

A large number of previous short-term studies reported an influence of the type of surgery on T2DM remission and a higher long-term rate of diabetes remission with RYGB than purely restrictive procedures [17, 24]. On the other hand, the Swedish Obese Subjects (SOS) study suggested that within a given weight change class, the changes in fasting insulin and HOMA-IR over 2 and 10 years are similar with banding, RYGB, and SG. (4) The low number of non–bypass procedures in our study, which makes it difficult to draw any definitive conclusions about these procedures.

This study has several limitations that must be acknowledged. Firstly, it is a retrospective study, and there is a significant number of patients who were lost to long-term follow-up in our cohort, potentially introducing bias into our patient selection.

Another weakness of this study is the imbalanced distribution of genders, with a higher prevalence of females. Additionally, our study population exhibited relatively good pre-operative glycemic control, reflected by a mean pre-operative HbA1c of 7.0%, which limits the generalization of our findings to patients with more decompensated diabetes. Furthermore, the clinical information in the first 2 years of follow-up was obtained through the medical records of our center, while the data of the 10 years after surgery was collected from electronic records of primary health care services, which could lead to information bias.

Despite these limitations, this study stands as a large single-center bariatric cohort with a 10-year follow-up, with a substantial number of patients, that is expected to contribute to the current literature on the long-term follow-up of bariatric patients.

## Conclusion

In conclusion, MBS can induce a significant and sustainable remission and improvement of T2DM. Consistent with previous studies, our data suggests the importance of an early intervention, while patients with diabetes still have a good glycemic control with a lower number of anti-diabetic drugs, in order to achieve “surgical cure” of T2DM. Longer follow-up reports and prospective, randomized controlled studies are important to confirm these findings.

## Data Availability

No datasets were generated or analysed during the current study.
